# ED_90_ of intravenous remimazolam for alleviating preoperative anxiety in children: a prospective dose-finding study

**DOI:** 10.3389/fmed.2026.1761997

**Published:** 2026-03-20

**Authors:** Xu-ming Zhu, Xiao-yong Wei, Qian-yu Guo, Pei-pei Hao, Le-ting Ji, Chang-sheng Li

**Affiliations:** Department of Anesthesiology, The Third Affiliated Hospital of Zhengzhou University, Zhengzhou, China

**Keywords:** biased coin design, children, intravenous drip, preoperative anxiety, remimazolam

## Abstract

**Background:**

Children’s minds are immature, making them more susceptible to severe anxiety when separated from their parents before surgery. Alleviating preoperative anxiety in children is essential for providing comfort-oriented healthcare. Remimazolam is a novel ultra-short-acting benzodiazepine. Existing studies have identified the effective dose for 50% of patients (ED_50_) when administering intravenous remimazolam to alleviate preoperative anxiety in children. However, the 90% effective dose (ED_90_) is clinically more meaningful. This study aims to determine the ED_90_ of intravenous remimazolam in alleviating preoperative anxiety in pediatric patients aged 1–6 years.

**Methods:**

From April to August 2025, pediatric patients undergoing elective surgery under general anesthesia were enrolled and stratified into two age groups: a younger group (YG, aged 1 to <4 years) and an older group (OG, aged 4–6 years inclusive). We employed the biased coin design (BCD) to determine target doses, defining a positive response as effective relief of preoperative anxiety [Parent Separation Anxiety Scale (PSAS) score < 3]. The initial dose for the first patient was 0.2 mg/kg. For subsequent pediatric patients, the dose was adjusted by increasing or decreasing by 0.05 mg/kg based on sedation response of the previous case. Isotonic regression and bootstrapping methods were used to estimate the ED_90_ and 90% confidence interval (CI), respectively.

**Results:**

Eighty children completed the study, 40 in YG group and 40 in OG group. Statistical analysis indicated that the ED_90_ (90% CIs) values for remimazolam used for alleviating preoperative anxiety in pediatric patients were 0.20 mg/kg (0.17–0.24) in the YG group and 0.15 mg/kg (0.11–0.17) in the OG group. Given that the 83% CIs for the ED_90_ showed no overlap between the YG group (0.18–0.24) and the OG group (0.12–0.17), the difference between the two groups is considered statistically significant.

**Conclusion:**

The ED_90_ of intravenous remimazolam for preoperative anxiolysis was 0.20 mg/kg in children aged 1 to <4 years and 0.15 mg/kg in those aged 4–6 years. Notably, older children require even lower weight-based doses.

**Clinical Trial Registration:**

https://www.chictr.org.cn/bin/project/edit?pid=252632, identifier ChiCTR2500098747.

## 1 Introduction

Children’s preoperative anxiety can reach as high as 50%–80% due to their immature physical and psychological development (1). This anxiety is heightened by separation from parents, significantly impacting their overall well-being (2, 3). With the advancement of patient-centered care, effectively alleviating preoperative anxiety has become a focal point for anesthesiologists. Standard methods for alleviating anxiety currently include non-pharmacological interventions (4), such as parental presence, videos or music, toys, and storybooks, along with pharmacological interventions like midazolam and dexmedetomidine. The effectiveness of non-pharmacological interventions is still uncertain. Oral midazolam has a slow onset of action (5), while intranasal dexmedetomidine may cause a burning sensation in the nose and a prolonged onset and prolonged drowsiness during recovery from anesthesia. Additionally, high doses of dexmedetomidine can lead to hypotension and bradycardia (6, 7). Remazolam is a novel ultra-short-acting benzodiazepine. When given intravenously, it is rapidly hydrolyzed by tissue esterases into inactive metabolites. The drug has a rapid onset, a short half-life, and does not accumulate in the body, allowing for swift recovery. It causes mild respiratory and circulatory depression with no injection pain, making it particularly advantageous for preoperative sedation (8). Currently, there is limited research on the use of remimazolam in pediatric populations. Previous studies have reported that the 50% effective dose (ED_50_) of intravenous remimazolam for preoperative sedation in pediatric patients aged 1–6 years is 0.15–0.17 mg/kg, with no significant age-related difference in sedative efficacy (9). However, the effective dose for 90% of patients (ED_90_) is of greater clinical importance, as it covers 90% of patients. Currently, no ED_90_ data are available for alleviating preoperative anxiety in this specific population. This study aims to determine the ED_90_ of intravenous remimazolam for alleviating preoperative anxiety in children aged 1–6 years, utilizing a biased coin design (BCD).

## 2 Methods

### 2.1 Study design and patients

This is a prospective, double-blind dose-response study approved by the Ethics Committee of the Third Affiliated Hospital of Zhengzhou University (Approval No.: 2024-421-01) and registered with the China Clinical Trial Registry on March 13, 2025 (ChiCTR2500098747). Using consistent inclusion and exclusion criteria, the two age groups were enrolled as independent cohorts during the same period. Sample size was calculated using the BCD method to estimate the ED_90_ of remimazolam for preoperative sedation, informed by a key review [Pace et al. (10)] and recent remimazolam dosage studies [Long et al. (11), Qu et al. (12)]. As the BCD method stabilizes ED_90_ estimates at 40 cases and stratified analysis by age (1–4 vs. 4–6 years) was required, 40 patients per cohort (total 80) were enrolled, providing sufficient data to estimate the ED_90_ and its 90% CI. Researchers obtained written informed consent from all participants’ guardians and encouraged children to participate in the study. Ultimately, 80 children participated in the study From April to August 2025.

### 2.2 Criteria for inclusion and exclusion

Inclusion criteria were: American Society of Anesthesiologists (ASA) physical status I–II, any sex, body mass index (BMI) of 13–20 kg/m^2^, and age either 1 to <4 years (younger group, YG) or 4–6 years inclusive (older group, OG).

Exclusion Criteria were: individuals with upper respiratory tract infections within the past 2 weeks; Anemia, malnutrition, or abnormal liver/kidney function; Severe cardiovascular disease; Intellectual disability; Neuropsychiatric disorders; History of allergy to anesthetic drugs; History of sedative-hypnotic medication use within the past 2 weeks; Parent-Child Separation Anxiety Scale (PSAS) score (The full PSAS scale is presented in Supplementary Table 1) <3; or guardians who refuse to participate in this study.

### 2.3 Research protocol

To ensure proper blinding throughout the study, the same anesthesiologist prepared the required dose of remimazolam in accordance with the study protocol. Another researcher, who was unaware of the dosage, assessed the success or failure of the PSAS score and collected data throughout the study. Neither the pediatric patients nor their guardians were informed of the administered dosage. All pediatric patients were instructed to fast from solid food for 8 h and from liquids for 2 h prior to surgery, and they received no preoperative medication. All pediatric patients had intravenous access established in the ward, accompanied by their parents. The children were escorted by their parents into the pre-anesthesia room of the operating suite. Prior to sedation, baseline characteristics, including age, gender, height, weight, and ASA status, were collected. During the pre-anesthesia period, pediatric patients undergo a Pediatric Separation Anxiety Scale (PSAS) assessment. Patients with a PSAS score of 3 or higher are identified as experiencing separation anxiety. These patients were accompanied by their parents and monitored non-invasively for blood pressure, heart rate (HR), and pulse oxygen saturation (SpO_2_), with baseline values for MAP, HR, and SpO_2_ recorded as T_0_. After administering remimazolam intravenously according to the trial protocol, a different anesthesiologist, who was unaware of the dosage, evaluated parental separation. Mean arterial pressure (MAP), HR, and SpO_2_ were recorded at 1 min (T_1_), 2 min (T_2_), and 3 min (T_3_) following the injection of remimazolam. Document any adverse reactions such as respiratory depression, hypoxemia, nausea/vomiting, hiccups, and allergic reaction, as well as drug reactions like smiling or panic that occur during the preoperative anti-anxiety period, which is defined as the time from the administration of pre-anesthesia medication until the induction begins in the operating room. This includes symptoms such as respiratory depression, nausea/vomiting, and hiccups, as well as drug reactions like smiling or panic. If the PSAS score remains three or higher after 3 min of observation, administer 1 mg/kg of propofol intravenously as rescue therapy.

The BCD method was employed to determine the target dose. Both YG Group and OG Group pediatric patients initially received an intravenous dose of 0.2 mg/kg of remimazolam. The sedation success of the previous patient determined the subsequent dose for each patient. Post-dosing at 3 min: if the preceding patient achieved a PSAS score of 3 or higher, it was defined as a negative outcome, and the subsequent patient received an increased dose by one gradient. Conversely, if the preceding patient’s PSAS score was less than 3 at the 3-min post-dosing mark (considered a positive outcome), the next patient’s dose was decided randomly via a computerized coin toss. A number between 1 and 100 was randomly selected. If the drawn number was 10 or lower, the next patient’s dose was reduced by one gradient. If the number was greater than 10, the dose remained unchanged. Adjacent dose gradients were set at 0.05 mg/kg, and this process continued until the 40th patient.

### 2.4 Data statistical analysis

Data were analyzed using the Statistical Package for the Social Sciences (SPSS, version 26.0), and graphs were generated using GraphPad Prism 10. For normally distributed continuous variables, results are presented as mean ± standard deviation (SD). Between-group comparisons were analyzed using the independent-samples *t*-test, while within-group comparisons were conducted with a repeated-measures analysis of variance (ANOVA). For skewed quantitative data, results were presented as median (interquartile range) [M(IQR)]. Intergroup comparisons were conducted using the Mann-Whitney U test. Categorical variables were presented as counts and/or percentages, with intergroup comparisons conducted using Pearson’s chi-square test. In this study, age was used as a stratification factor. The appropriate validation method described above was applied according to the data distribution characteristics to verify intergroup balance. *P* < 0.05 was considered statistically significant.

Due to patient variability, the assumption that remimazolam’s effect increases monotonically with dose is not always consistent. Therefore, the Proportional Adjacent Violators Algorithm (PAVA) was employed to ensure monotonic response rates (10, 13). Isotonic regression analysis was performed using the “R” package (R version 4.4.0) to fit dose-response curves for two remimazolam groups and to obtain the ED_90_ for remimazolam in alleviating preoperative anxiety. The 90% CI was calculated via bootstrapping with 2000 repeated samples (10). Drawing upon prior literature (14), we utilized the overlapping CI method to assess statistical differences between two sets of ED_90_ estimates. Specifically, non-overlapping 83% CIs were interpreted as evidence of a statistically significant difference between the groups.

## 3 Results

### 3.1 Demographics

**FIGURE 1:**
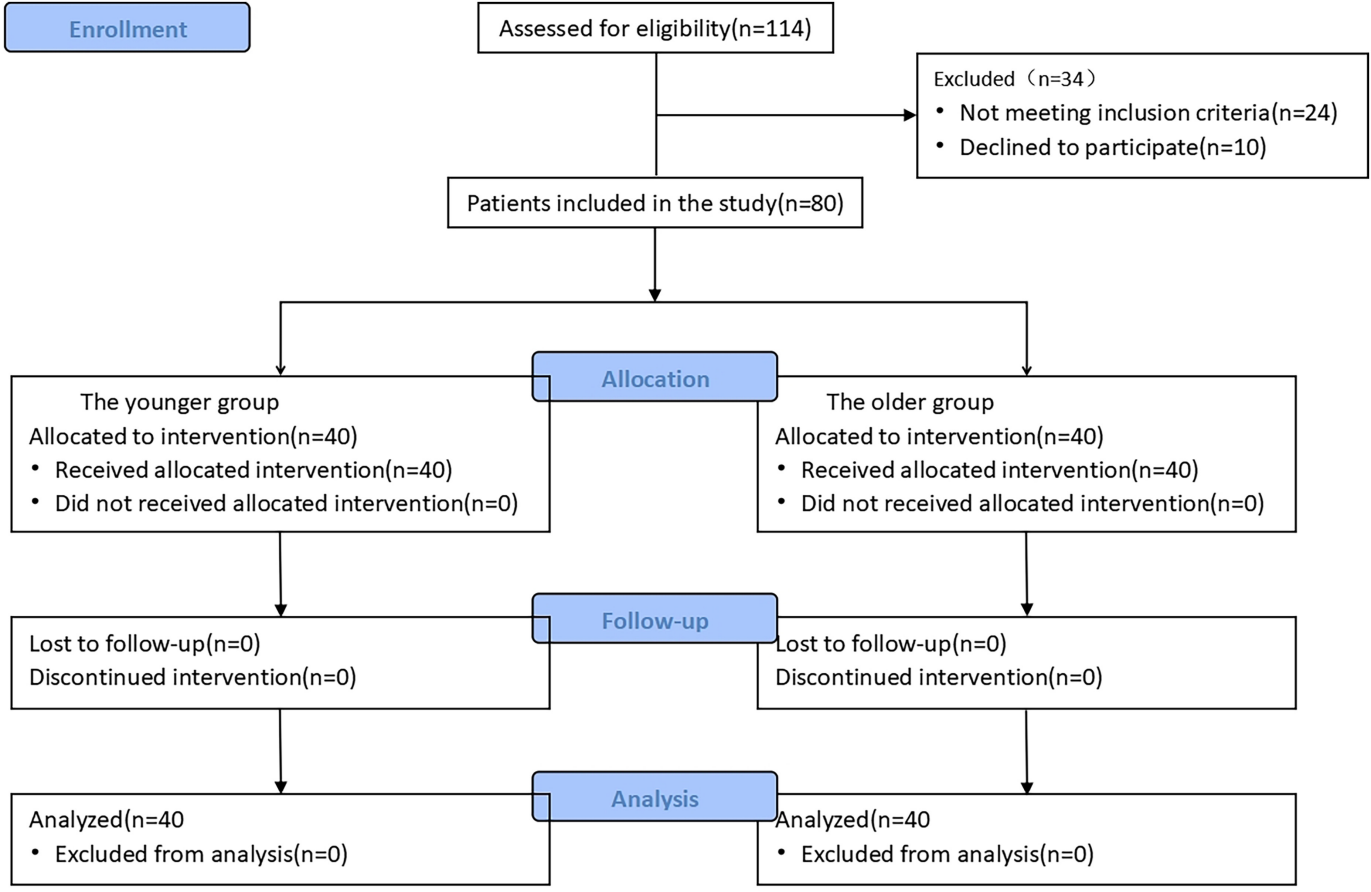
CONSORT flow diagram of this study.

We assessed a total of 114 children who underwent laparoscopic high ligation of the processus vaginalis between April and August 2025. Of these, 80 patients provided informed consent and completed the study. This group consisted of 40 children in the younger group (YG group) and 40 in the older group (OG group) (Figure 1). Demographic data are shown in Table 1. Statistically significant differences were found between the two groups in terms of age, height, and body weight (*P* < 0.05). Other general characteristics did not show statistically significant differences (*P* > 0.05).

**TABLE 1:** Demographic data.

Index	YG group (*n* = 40)	OG group (*n* = 40)	*P*-value
Age (year)	2.0 (2.0)	4.7 (0.8)	<0.001
Height (cm)	87.3 ± 10.2	110.1 ± 6.6	<0.001
Weight (kg)	12.1 (4.8)	19.5 (3.8)	<0.001
BMI (kg/m^2^)	16.7 (2.1)	16.1 (1.6)	0.086
Sex (male/female)	36/4	30/10	0.077

BMI stands for body mass index.

### 3.2 ED_90_

**FIGURE 2:**
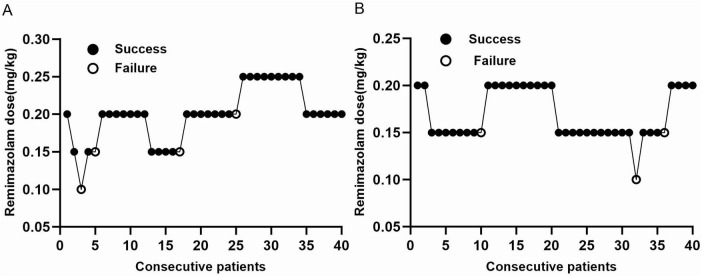
Reaction trajectory of remimazolam dose in the YG and OG groups. **(A)** YG group. **(B)** OG group.

Of the 80 pediatric patients enrolled in the study, 36 in YG Group and 37 in OG Group showed positive responses to anxiolysis. The response trajectories for the positive and negative sequences of preoperative anxiolysis achieved with the target dose of remimazolam are illustrated in Figure 2. Isotonic regression yielded ED_90_ estimates of 0.20 mg/kg (YG groups) and 0.15 mg/kg (OG groups) for preoperative remimazolam anxiolysis; the corresponding 90% CIs, derived from 2000 bootstrap resamples, were (0.17–0.24) mg/kg and (0.11–0.17) mg/kg. Given that the 83% CIs for the ED_90_ showed no overlap between the YG group (0.18–0.24 mg/kg) and the OG group (0.12–0.17 mg/kg), the difference between the two groups is considered statistically significant, as shown in Table 2.

**TABLE 2:** Comparison of effective preoperative anxiolytic doses between two groups of pediatric patients.

Group	YG group	OG group
ED_90_ (mg/kg)	0.20	0.15
90% CI (mg/kg)	0.17, 0.24	0.11, 0.17
83% CI (mg/kg)	0.18, 0.24	0.12, 0.17

### 3.3 Hemodynamics

**FIGURE 3:**
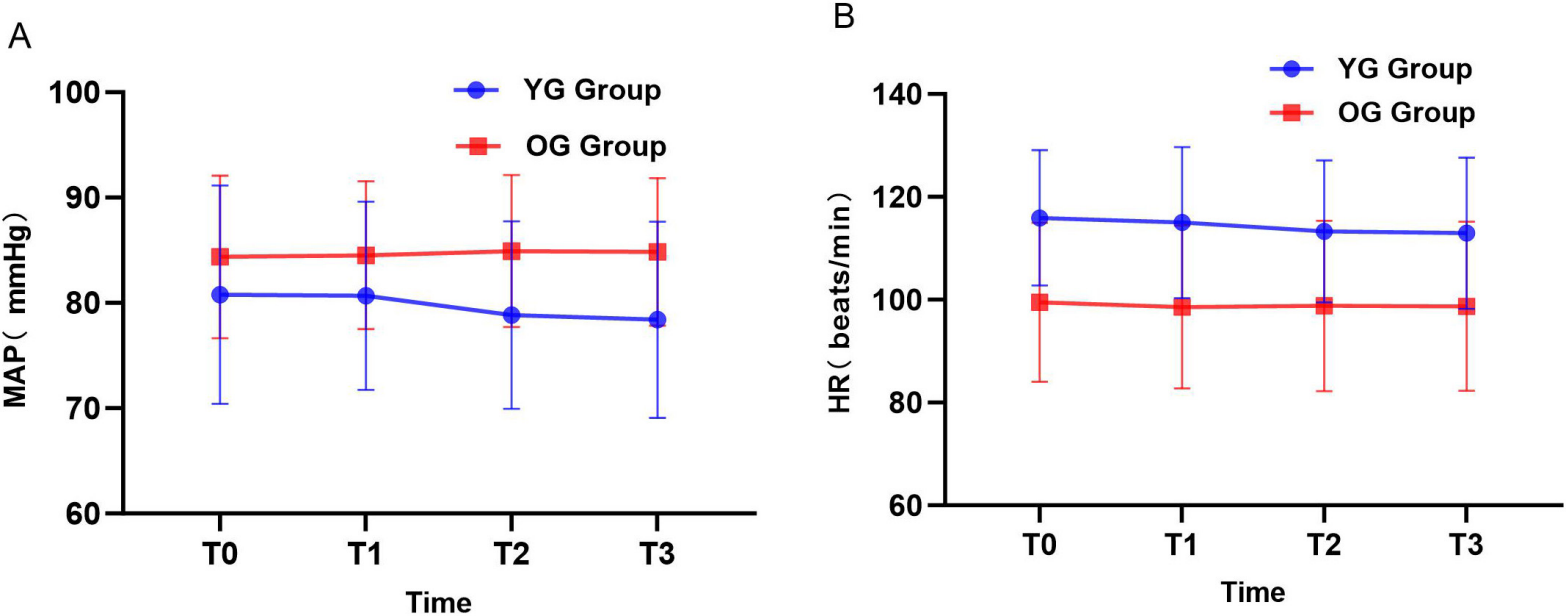
Changes in MAP and HR at different time points in the YG and OG groups. **(A)** YG group. **(B)** OG group.

Figure 3, display the MAP and HR values for the two pediatric patient groups at different time points. Compared with T_0_, there were no statistically significant differences in MAP and HR between the two groups at T_1_, T_2_, and T_3_ (*P* > 0.05).

### 3.4 Adverse events

Neither group of pediatric patients exhibited respiratory depression, hypoxemia, nausea, vomiting, hiccups, or allergic reactions.

## 4 Discussion

Anxiety is a common negative emotion in children before surgery and remains a major concern for anesthesiologists. Research indicates (15, 16) that preoperative anxiety in children is closely related to their age. Younger patients, due to their lower cognitive abilities, tend to experience higher levels of anxiety before surgery. Specifically, children under 6 years old have significantly higher preoperative anxiety scores compared to older children (16). In this study, children were categorized into younger and older groups using 4 years of age as the cutoff. This classification aligns with the approach employed in Zhang et al.’s study on the ED_50_ of esketamine for preoperative anxiety (17). A randomized controlled trial (18) conducted in Pakistan also found that the incidence of preoperative anxiety in children aged 2–4 years (61.3%–66%) was significantly higher than that in children aged 5–12 years (32.1%–41.5%). In a study by Zhang et al. (19) involving children undergoing burn surgery, preoperative anxiety scores were found to be significantly higher in the 1–3-years-old group (<4 years) than in the 4–14-years-old group. Although these studies vary in their subjects and contexts, they consistently suggest that age 4 may represent a potential critical threshold for research on preoperative anxiety among preschool children. Accordingly, this study performed a subgroup analysis using 4 years of age as the cutoff.

Remimizolam is an ultra-short-acting benzodiazepine that targets gamma-aminobutyric acid (GABA) A receptors (GABAA) to induce sedative and hypnotic effects. It is quickly metabolized by nonspecific esterases in the bloodstream. Compared with midazolam, remimazolam has a faster distribution phase, a shorter terminal half-life, and a higher clearance rate (20). Previous studies have demonstrated that the pharmacokinetics of remimazolam after intravenous infusion in pediatric patients are similar to those in adults, exhibiting rapid onset and offset of action, and no accumulation during prolonged infusion (21). Recent studies indicate that remimazolam can be used safely and effectively for the induction of general anesthesia, the prevention of agitation, and sedation during magnetic resonance imaging in pediatric patients (22–24). Intranasal administration of remimazolam effectively reduces preoperative anxiety in pediatric patients (11, 25). However, data regarding the effective dosage of intravenous remimazolam for alleviating preoperative anxiety in pediatric patients with established venous access remain limited. Our findings indicate that the ED_90_ values for preoperative anxiolysis with remimazolam were 0.20 mg/kg in YG group and 0.15 mg/kg in OG group. A statistically significant difference in the effective dose between the two age groups was demonstrated by the non-overlapping 83% CIs for the two ED_90_ values. This finding suggests that older children (aged 4 years and above) require lower doses of remimazolam for preoperative anxiolysis, potentially due to higher levels of neurodevelopment and educational attainment in the older age group (10, 23). The specific mechanisms behind this observation warrant further investigation. The results of the present study differ from those reported by Chen et al. (9), who employed an up-down method to stratify pediatric patients aged 1–6 years into five 1-year interval subgroups. In their study, the ED_50_ of remimazolam for preoperative sedation was measured at 0.15–0.17 mg/kg, with no significant differences detected among the subgroups. This apparent discrepancy in conclusions does not signify a contradiction, but rather arises from systematic differences in study design. The previous study concentrated on ED_50_ values, which reflect baseline effects, and incorporated detailed age stratification, whereas the present study determined ED_90_ values to address clinical requirements for high sedation success rates. By grouping participants according to developmental stage and employing a biased-coin sequential method, this study was able to more effectively capture age-related dose variations. Furthermore, the ED_90_ dose of remimazolam identified for alleviating preoperative anxiety in pediatric patients in this study was substantially lower than the doses previously reported for induction of general anesthesia in children aged 1–6 years (ED_50_: 0.41–0.42 mg/kg; ED_95_: 0.57 mg/kg) (26). This marked discrepancy in dosage primarily arises from the distinct clinical objectives of drug administration: induction of general anesthesia requires achieving a profound anesthetic state with loss of consciousness to satisfy surgical demands, while alleviation of preoperative anxiety only necessitates mild to moderate sedation to promote patient cooperation. The gradient in dosage thresholds between these two situations offers valuable reference points for individualized clinical dosing. Synthesizing the results of this study with prior research enables the development of comprehensive, evidence-based recommendations for selecting individualized remimazolam dosages in pediatric patients across various clinical indications and dose-response profiles.

The biased coin design method adopted in this study uses a probability advantage ratio Γ (10), with a target probability of 0.90. After a positive response from a patient, the subsequent patient does not receive a direct dose reduction; instead, they undergo biased coin randomization. The biased coin design possesses distinct methodological characteristics compared to data generated by the traditional Dixon sequential method. The Dixon sequential method primarily focuses on ED_50_ estimation, facilitating precise calculation of ED_50_ but resulting in considerable deviation when determining ED_90_. In contrast, the biased coin design centers on ED_90_, enabling more accurate measurements of ED_90_ with reduced statistical deviation and greater precision (27, 28). Accordingly, this study utilized the biased coin design to determine the ED_90_ of remimazolam, as ED_90_ values are more appropriate than ED_50_ values for informing clinical anesthetic dosing decisions. Based on preliminary studies and literature on pediatric preoperative sedation (29), we set the remimazolam dose for the first patient at 0.2 mg/kg. The initial dose per unit of body weight may be relatively higher than those studied in adults (30, 31). Preschool children have a higher water content, which leads to a larger volume of distribution for water-soluble drugs. As a result, higher doses of remimazolam may be necessary for pediatric patients.

In the pre-anesthesia room, pediatric patients reacted to intravenous administration of remimazolam with responses such as smiling, quietness, crying, and agitation. YG group’s primary reactions included smiling (22.50%), quietness (62.5%), initial fear followed by smiling (5%), fear (2.5%), and crying (7.5%); OG group’s primary reactions included smiling (32.50%), quietness (60%), fear (5%), and crying (2.5%). The child’s display of fear and anxiety may result from either a high-speed injection rate or an insufficient dose of sedative medication. In two cases within YG group, the child initially showed signs of fear and anxiety before transitioning to smiling. This change in expression may be linked to an initially slow injection rate, similar to the reaction observed with an inadequate medication dosage. Once the appropriate dose began to take effect, the child’s expression changed to a smile. Reports indicate that remimazolam nasal spray also induces a smiling response (22). The exact mechanism behind this effect is not yet understood and requires further investigation. No cases of respiratory depression, hypoxemia, nausea, or vomiting were observed in any of the pediatric patients. The hemodynamic effects of remimazolam are minimal. Our study found that HR and MAP in both groups fluctuated little compared to baseline prior to induction of anesthesia, with changes not exceeding 20% of baseline values, which is generally considered acceptable.

This study has certain limitations. First, the sample size of 40 patients per group was determined by referencing similar studies by Long et al. (11) (Frontiers in Medicine, 2023) and Qu et al. (12) (Frontiers in Pharmacology, 2023), which used a biased coin design to estimate remimazolam ED values. However, this sample size is below the optimal recommendation of 50–60 patients for ED_90_ estimation, as suggested by Oron et al. (32) (Anesthesiology, 2022). Consequently, the ED_90_ estimate for remimazolam in this study should be considered preliminary and of limited precision. Second, the dose intervals for remimazolam in this study were established at 0.05 mg/kg, resulting in a limited number of gradient levels. While this approach encompassed the core effective range of ED_90_, as determined by preliminary experiments and provided sufficient reliability for dose estimation, it did not allow for a detailed characterization of the dose-response curve. Future investigations may utilize narrower intervals of 0.02–0.03 mg/kg to further refine the dose-response data. Third, this study included only pediatric patients classified as ASA I–II, and the dosage characteristics for special populations remain undetermined.

In summary, intravenous administration of remimazolam effectively alleviates preoperative anxiety in pediatric patients. Notably, children younger than 4 years required a significantly higher weight-based dose of medication compared to those aged 4–6 years. While the sample size was limited, these findings may serve as a preliminary reference for clinical practice. Further validation with larger sample sizes is warranted.

## Data Availability

The original contributions presented in this study are included in this article/Supplementary material, further inquiries can be directed to the corresponding authors.

## References

[1] Liang Y Huang W Hu X Jiang M Liu T Yue H et al. Preoperative anxiety in children aged 2-7 years old: a cross-sectional analysis of the associated risk factors. *Transl Pediatr.* (2021) 10:2024–34. 10.21037/tp-21-215 34584872 PMC8429856

[2] Kalogianni A Almpani P Vastardis L Baltopoulos G Charitos C Brokalaki H. Can nurse-led preoperative education reduce anxiety and postoperative complications of patients undergoing cardiac surgery? *Eur J Cardiovasc Nur.* (2016) 15:447–58. 10.1177/1474515115602678 26304701

[3] Yan Ying P Shen Ling L Xiao Han P Yun Bo X Xin T Guo Yan L et al. Incidence and risk factors associated with negative postoperative behavioral changes in children undergoing painless gastroscopy. *BMC Pediatr.* (2023) 23:371. 10.1186/s12887-023-04187-8 37474961 PMC10360286

[4] Chen H Zhang J Li S Zhang H Wei L. Non-pharmacological interventions for preoperative anxiety in children: a systematic review and network meta-analysis. *J Clin Nurs.* (2025) 34:1493–507. 10.1111/jocn.17582 39763216

[5] Wang J Zeng J Zhao N Chen S Chen Z Liao J. Intranasal esketamine combined with oral midazolam provides adequate sedation for outpatient pediatric dental procedures:a prospective cohort study. *Int J Surg.* (2023) 109:1893–9. 10.1097/JS9.0000000000000340 37288546 PMC10389564

[6] Cai Y Wang C Fang Y Ma H Gao Y Wang Z et al. Preoperative anxiolytic and sedative effects of intranasal remimazolam and dexmedetomidine: a randomized controlled clinical study in children undergoing general surgeries. *Drug Des Devel Ther.* (2024) 18:1613–25. 10.2147/DDDT.S461122 38774484 PMC11108072

[7] Hermans K Ramaekers L Toelen J Vanhonsebrouck K Allegaert K. Intranasal dexmedetomidine as sedative for medical imaging in young children: a systematic review to provide a roadmap for an evidence-guided clinical protocol. *Children.* (2022) 9:1310. 10.3390/children9091310 36138619 PMC9498011

[8] Pieri M D’Andria Ursoleo J Di Prima A Bugo S Barucco G Licheri M et al. Remimazolam for anesthesia and sedation in pediatric patients: a scoping review. *J Anesth.* (2024) 38:692–710. 10.1007/s00540-024-03358-w 38844707

[9] Chen Y Zhang W Ma J Liu W Song X Chen X. Median effective dose of remimazolam for preoperative sedation in pediatric patients of different ages. *Chinese J Anesthesiol.* (2024) 44:1207–10. 10.3760/cma.j.cn131073.20240227.01011

[10] Pace Nathan L Stylianou Mario P. Advances in and limitations of up-and-down methodology: a précis of clinical use, study design, and dose estimation in anesthesia research. *Anesthesiology.* (2007) 107:144–52. 10.1097/01.anes.0000267514.42592.2a 17585226

[11] Long X Wen L Yang H Zhu G Zhang Q Jiang J et al. ED95 of remimazolam in nasal administration for attenuating preoperative anxiety in children. *Front Med.* (2023) 10:1253738. 10.3389/fmed.2023.1253738 37680615 PMC10482406

[12] Qu L Liu M Ouyang R Li T Long D Jiang Y et al. Determination of the 95% effective dose of remimazolam tosylate in anesthesia induction inhibits endotracheal intubation response in senile patients. *Front Pharmacol.* (2023) 14:1136003. 10.3389/fphar.2023.1136003 37324498 PMC10266225

[13] Borrat X Valencia J Magrans R Gimenez-Mila M Mellado R Sendino O et al. Sedation-analgesia with propofol and remifentanil: concentrations required to avoid gag reflex in upper gastrointestinal endoscopy. *Anesth Analg.* (2015) 121:90–6. 10.1213/ANE.0000000000000756 25902320

[14] Liu L Drzymalski D Xu W Zhang W Xiao F. Dose dependent reduction in median effective concentration (EC50) of ropivacaine with adjuvant dexmedetomidine in labor epidural analgesia: an up-down sequential allocation study. *J Clin Anesth.* (2021) 68:110115. 10.1016/j.jclinane.2020.110115 33142249

[15] Getahun A Endalew N Mersha A Admass B. Magnitude and factors associated with preoperative anxiety among pediatric patients: cross-sectional study. *Pediatric Health Med Ther.* (2020) 11:485–94. 10.2147/PHMT.S288077 33364873 PMC7751437

[16] Mathew P Gopinath A Gupta A Yaddanapudi S Panda N Kohli A. Assessment of potential predictors affecting preoperative anxiety in Indian children- A prospective observational study. *J Anaesth Clin Pharm.* (2023) 39:279–84. 10.4103/joacp.joacp_371_21 37564837 PMC10410033

[17] Zhang B Yang Y Jia J Meng F Zhang J. Median effective dose of esketamine for preoperative sedation in pediatric patients of different ages. *Chinese J Anesthesiol.* (2022) 42:320–2. 10.3760/cma.j.cn131073.20211212.00315

[18] Ali M Khan M Salim B. Comparing pharmacological and nonpharmacological interventions for alleviating preoperative anxiety in pediatric surgical patients: a randomized controlled trial in Pakistan. *Cureus.* (2025) 17:e82502. 10.7759/cureus.82502 40385813 PMC12085961

[19] Wu Y Zuang L Wang F. Risk factors for preoperative anxiety and postoperative pain in children undergoing burn surgery and their relationship. *Shanghai Med J.* (2023) 46:840–8. 10.19842/j.cnki.issn.0253-9934.2023.12.007

[20] Murrell D Harirforoosh S. Clinical pharmacokinetics, pharmacodynamics, and drug interactions of remimazolam. *Eur J Drug Metab Pharmacokinet.* (2025) 50:449–60. 10.1007/s13318-025-00963-2 40965620 PMC12540523

[21] Gao Y Ihmsen H Hu Z Sun W Fang Y Wang Z et al. Pharmacokinetics of remimazolam after intravenous infusion in anaesthetised children. *Brit J Anaesth.* (2023) 131:914–20. 10.1016/j.bja.2023.08.019 37739904

[22] Cai Y Dong L Zhong J Lin Z Chen C Zhu L et al. ED50 and ED95 of remimazolam for loss of consciousness in young children: a dose-finding study for induction of anaesthesia. *Br J Anaesth.* (2025) 134:1709–16. 10.1016/j.bja.2025.02.004 40107902 PMC12106876

[23] Wu Y Wang F Zhu K Ling L Zhang W. A randomized controlled study of remimazolam in preschool children undergoing adenotonsillectomy. *Front Pharmacol.* (2025) 16:1678650. 10.3389/fphar.2025.1678650 41158123 PMC12558047

[24] Shi H Zhu J Liu J Wang L Yan J. Comparative evaluation of remimazolam besylate versus propofol for pediatric MRI sedation: safety, recovery, and adverse event profiles. *Int J Gen Med.* (2025) 18:6315–25. 10.2147/IJGM.S542191 41132308 PMC12543097

[25] Ni M Jin Y Wu Q Zhang N Tian J Li J et al. Effective dose of intranasal remimazolam for preoperative sedation in preschool children: a dose-finding study using Dixon’s up-and-down method. *Front Pharmacol.* (2024) 15:1372139. 10.3389/fphar.2024.1372139 38572430 PMC10987844

[26] Cai Y Dong L Zhong J Lin Z Chen C. ED50 and ED95 of remimazolam for loss of consciousness in young children: a dose-finding study for induction of anaesthesia. *Br J Anaesth.* (2025) 134:1709–16. 10.1016/j.bja.2025.02.004 40107902 PMC12106876

[27] Görges M Zhou G Brant R Ansermino J. Sequential allocation trial design in anesthesia: an introduction to methods, modeling, and clinical applications. *Pediatr Anesth.* (2017) 27:240–7. 10.1111/pan.13088 28211193

[28] Iasonos A Gönen M Bosl G. Scientific review of phase i protocols with novel dose-escalation designs: how much information is needed? *J Clin Oncol.* (2015) 33:2221–5. 10.1200/JCO.2014.59.8466 25940721 PMC4962621

[29] Wu M Yang F Ma X Cai N. Comparison of clinical effects and safety of remidazolam and esketamine for preoperative sedation in children. *J Southern Med Univer.* (2023) 43:2126–31. 10.12122/j.issn.1673-4254.2023.12.18 38189400 PMC10774111

[30] Tan H Lou A Wu J Chen X Qian X. Determination of the 50% and 95% effective dose of remimazolam combined with propofol for intravenous sedation during day-surgery hysteroscopy. *Drug Des Devel Ther.* (2023) 17:1753–61. 10.2147/DDDT.S406514 37333966 PMC10276603

[31] Lu Z Zhou N Li Y Yang L Hao W. Up-down determination of the 90% effective dose (ED90) of remimazolam besylate for anesthesia induction. *Ann Palliat Med.* (2022) 11:568–73. 10.21037/apm-22-89 35249335

[32] Oron A Souter M Flournoy N. Understanding research methods: up-and-down designs for dose-finding. *Anesthesiology.* (2022) 137:137–50. 10.1097/ALN.0000000000004282 35819863

