# Correction: ED_90_ of intravenous remimazolam for alleviating preoperative anxiety in children: a prospective dose-finding study

**DOI:** 10.3389/fmed.2026.1841001

**Published:** 2026-04-16

**Authors:** Xu-ming Zhu, Xiao-yong Wei, Qian-yu Guo, Pei-pei Hao, Le-ting Ji, Chang-sheng Li

**Affiliations:** Department of Anesthesiology, The Third Affiliated Hospital of Zhengzhou University, Zhengzhou, China

**Keywords:** biased coin design, children, intravenous drip, preoperative anxiety, remimazolam

In the published article, there was an error in the author list: the equal first authorship of Xu-ming Zhu and Xiao-yong Wei was not marked with the standard dagger symbol (†). The corrected author list appears below.

Xu-ming Zhu^†^, Xiao-yong Wei^†^, Qian-yu Guo, Pei-pei Hao, Le-ting Ji^*^ and Chang-sheng Li^*^

^†^These authors share first authorship.

The original article has been updated.

