# Supplementary material for: ED90 of intravenous remimazolam for alleviating preoperative anxiety in children: a prospective dose-finding study
Source: Front Med (Lausanne). 2026 Mar 20;13:1761997. doi: 10.3389/fmed.2026.1761997 (PMC13036471; doi:10.3389/fmed.2026.1761997)
Supplement: Supplementary file 1 [file Table_1.doc]

Supplementary Table 1 Parental Separation Anxiety Scale(PSAS)

| **PSAS scores** | **PSAS 1 and 2：successful separation from the parents/guardians** |
| --- | --- |
| 1 | easily separated |
| 2 | Whimpering but easily consoled |
| 3 | Crying and difficult to console |
| 4 | Crying and clinging to parents |
